# Blood brain barrier permeability and astrocyte-derived extracellular vesicles in children with juvenile idiopathic arthritis: a cross-sectional study

**DOI:** 10.1186/s12969-024-00984-2

**Published:** 2024-04-26

**Authors:** Lillemor Berntson, Andreas Elfving, Alice Gabrielsson Samuelsson, Anders Öman, Fariborz Mobarrez

**Affiliations:** 1 Department of Women’s and Children’s Health, Uppsala University, Uppsala, Sweden; 2 Department of Medical Sciences, Clinical Chemistry, Uppsala University, Uppsala, Sweden

**Keywords:** Juvenile idiopathic arthritis, Extracellular vesicles, Astrocytes, S100B, Blood brain barrier

## Abstract

**Background:**

Juvenile idiopathic arthritis (JIA) is the most prevalent rheumatic disease in children, and the inflammatory process is widely studied, primarily characterized by its impact on joint health. Emerging evidence suggests that JIA may also affect the central nervous system (CNS). This study investigates the potential CNS involvement in JIA by analyzing the presence of astrocyte-derived extracellular vesicles (EVs) and the S100B protein in plasma, both of which are indicative of astrocyte activity and blood-brain barrier (BBB) integrity.

**Methods:**

EDTA plasma from 90 children diagnosed with JIA and 10 healthy controls, matched by age and gender, was analyzed for extracellular vesicles by flow cytometric measurement. Astrocyte-derived EVs were identified using flow cytometry with markers for aquaporin 4 (AQP-4) and glial fibrillary acidic protein (GFAP). Levels of the S100B protein were measured using a commercial ELISA. Disease activity was assessed using the Juvenile Arthritis Disease Activity Score (JADAS27, 0–57), and pain levels were measured using a visual analogue scale (VAS, 0–10 cm).

**Results:**

Our analyses revealed a significantly higher concentration of astrocyte-derived EVs in the plasma of children with JIA compared with healthy controls. Furthermore, children with JADAS27 scores of 1 or higher exhibited notably higher levels of these EVs. The S100B protein was detectable exclusively in the JIA group.

**Conclusion:**

The elevated levels of astrocyte-derived EVs and the presence of S100B in children with JIA provide evidence of BBB disruption and CNS involvement, particularly in those with higher disease activity. These findings underscore the importance of considering CNS health in the comprehensive management of JIA. Further research is required to elucidate the mechanisms behind CNS engagement in JIA and to develop treatments that address both joint and CNS manifestations of the disease.

**Supplementary Information:**

The online version contains supplementary material available at 10.1186/s12969-024-00984-2.

## Background

Juvenile idiopathic arthritis (JIA) is the most prevalent form of chronic arthritis in children and encompasses a variety of autoimmune and partly autoinflammatory conditions with onset before the age of sixteen [1, 2]. Characterized by persistent synovial inflammation, JIA leads to joint pain, swelling, and a risk of long-term disability and growth disturbances. The complexity of JIA’s etiology involves a dynamic interplay between genetic, environmental, and immunological factors, with current research efforts expanding our understanding of these interactions [3–6].

While the articular manifestations of JIA are well-documented, the potential involvement of the central nervous system (CNS) remains a relatively uncharted domain. The CNS implications in JIA are not as clearly defined as the joint-related symptoms, with limited information available on the extent and impact of neurological involvement. Children with JIA experience fatigue that exceeds what you find in healthy children and they often have persistent pain despite treatment [7, 8]. The increased risk of mental comorbidities is also an example of manifestations in which an inflammatory influence on the CNS may possibly contribute [9]. Recent studies have begun to shed light on this aspect, revealing that systemic inflammation in JIA may extend to the CNS, leading to alterations in brain structure and function, and suggesting a broader impact of the disease than previously recognized [10].

The blood brain barrier (BBB) is a critical structure formed by brain endothelial cells that serves to protect the brain from fluctuations in plasma composition and circulating neuroactive substances [11]. The integrity of the BBB is largely attributed to the complex tight junctions between endothelial cells, which are significantly influenced by the surrounding astrocytic glia. Astrocytes are central to the maintenance and modulation of the BBB, contributing to its selective permeability and response to physiological and pathological stimuli [12].

Extracellular vesicles (EVs) are cell released vesicles that regulate biological processes through cellular signaling, for example from astrocytes. Two EVs originating from astrocytes are aquaporin 4 (AQP-4) and glial fibrillary acidic protein (GFAP). The AQP-4 is a water channel in the brain localized on the axon terminals (end feet) of astrocytes which are in contact with the blood vessels of the BBB. It regulates water permeability and is an adhesion molecule involved in among other things, neuroexcitation, synaptic plasticity and cell migration [13]. Glial fibrillary acidic protein (GFAP), is the intermediate filament protein in astrocytes, also called the nanofilament, a part of the cytoskeleton of astrocytes [14]. GFAP proteins and products are released in biofluids and can act as biomarkers [15].

Astrocyte-derived EVs are increasingly recognized as important mediators of cell-to-cell communication within the CNS, capable of carrying proteins, lipids, and RNA between cells. These vesicles are implicated in various aspects of brain function and pathology, including the modulation of BBB permeability. The presence of astrocyte-derived EVs in the blood, which express aquaporin-4 and GFAP as identified in our previous work [16], indicates that these EVs could serve as markers for BBB dysregulation. This suggests that astrocytes may release EVs into the bloodstream as a response to systemic inflammatory diseases. Furthermore, the astrocytic protein S100B has been identified as a potential biomarker for CNS involvement, with elevated levels in peripheral blood observed in conditions associated with BBB disruption [17].

This study aims to elucidate the role of astrocyte-derived EVs and S100B in the context of BBB integrity in JIA. By quantifying these biomarkers and correlating them with clinical measures of disease activity, we seek to uncover the potential mechanisms by which systemic inflammation in JIA may affect CNS function and BBB permeability. This research may provide new insights into the CNS implications of JIA and highlight the importance of considering neurological health in the comprehensive management of this disease.

## Methods

### Study population

We included 90 children with JIA attending the Unit of Pediatric Rheumatology, Uppsala University Hospital, between 2016 and 2022 after varying duration of disease. The participants were classified according to the International League of Association for Rheumatology criteria [18].

We also included blood samples of 10 healthy children matched for gender and age, admitted for minor surgery, during the same time period. Exclusion criteria were medication for any disease, presence of any inflammatory disease, diabetes or any atopic disease with continuous medication or special diet because of intolerance. The samples were drawn pre-operatively.

Disease activity was assessed using the JADAS27. This composite score comprises a joint count (0–27 active joints), patient-reported global assessment of well-being on a visual analogue scale (VAS) (0–10 cm), assessed by a parent if the child is ≤ 9 years old, physician’s global assessment of disease activity on VAS (0–10 cm) and normalized erythrocyte sedimentation rate ((E-SR (mm/h) -20)/10) to a scale (0–10). The maximum total score of JADAS27 is 57 [19]. For scoring of pain we used a VAS (0–10 cm).

### Sample preparation

Venous blood samples were collected into K2-EDTA tubes and then centrifuged at 1500 x g for 10 min at room temperature (RT). The resulting plasma was aliquoted and stored at -70 °C. For the 90 children with JIA the samples were frozen after a median of 2.0 h (min–max 0.8–3.7), and for the controls after a median of 1.8 h (min-max 0.8–2.7). There was no statistical difference in time between sampling and centrifugation comparing patients and controls *p* = 0.22 (Independent-Samples Mann-Whitney U Test).

### Flow cytometric measurement of astrocyte-derived EVs

**Fig. 1:**
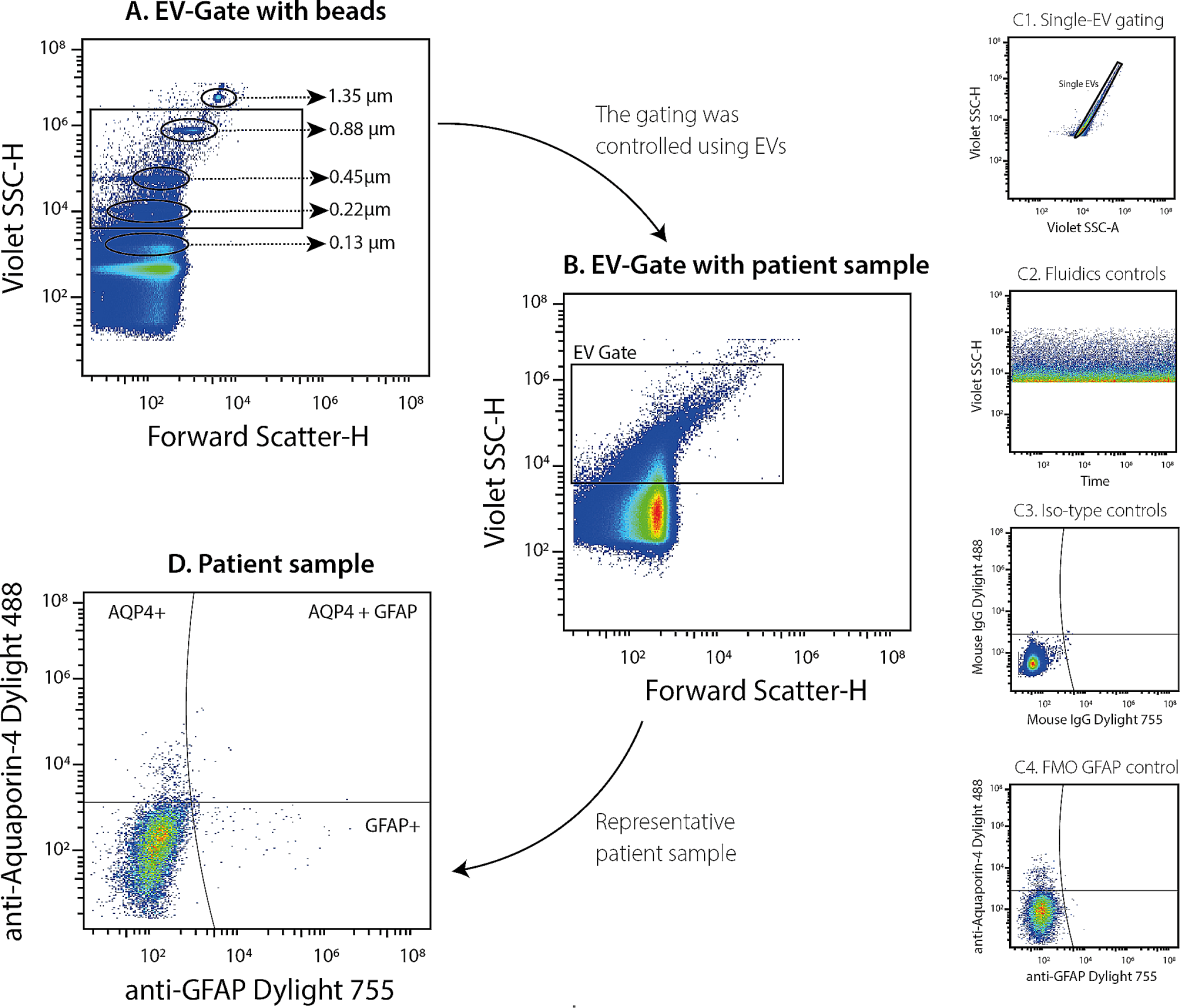
Representative plots of flow cytometric analysis of extracellular vesicles (EVs). (**A**) Dot-plot of Nano fluorescent Yellow Particles used to determine the EV gate. (**B**) Gating strategy for all EVs based on size and complexity. (C1-4) Beside size and complexity, samples were controlled for single gating (C1 and C2). Unstained EVs, Iso-type controls, single fluorochrome stained EVs, and EVs stained as fluorescence-minus-one (FMO) were used to fine tune the panel (C4). Representative sample of an JIA patient demonstrating EVs labeled with anti-Aquaporin-4 Dylight 488 and anti-GFAP Dylight 755

The frozen plasma samples (JIA or controls) were thawed in water bath for approximately 5 min at 37 °C prior to analysis. An EV-enriched pellet was isolated from EDTA-anticoagulated plasma by centrifugation at 2000 x g for 20 min at RT. The upper supernatant was transferred to new tubes and further centrifuged at 20 800 x g for 45 min at RT. The supernatant from this second centrifugation was discarded, and the remaining EV-enriched pellet was reserved for flow cytometric analysis. A 20 µL sample of the pellet was placed into a 96-well plate and incubated for 20 min in the dark with 5 µL of anti-Aquaporin-4 Dylight 488 and 5 µL of anti-GFAP Dylight 755 (Abcam, Cambridge, UK). The samples were then diluted with 120 µL of CytoFLEX Sheath Fluid (Beckman Coulter, Brea, CA, USA) prior to analysis by a CytoFLEX flow cytometer (Beckman Coulter). To establish the gating for EV analysis, Nano fluorescent Yellow Particles of 0.13 μm, 0.22 μm, 0.45 μm, 0.88 μm, and 1.35 μm (Spherotech, Lake Forest, IL, USA) were utilized, detecting a size range of approximately 0.2 μm to 1.0 μm (Fig. 1A, B, D). The lower EV gate was set at the 0.2 μm beads to avoid the significant background noise present around 0.13 μm, which could affect accurate measurements. Additionally, the gating strategy was verified using labeled and unlabeled EVs, as beads and EVs have different refractive indices. Unstained EVs, isotype controls, single fluorochrome-stained EVs, and EVs stained as fluorescence-minus-one (FMO) controls were employed to calibrate the instrument (Fig. 1). The instrument’s threshold was set to violet side scatter. Astrocyte-derived EVs were identified as EVs expressing both GFAP and aquaporin-4. Samples from patients and controls were analyzed at the same occasion. Results are presented as the number of EVs per microliter of plasma, derived from the 20 µL pellet post high-speed centrifugation. The intra- and interassay coefficients of variation for the flow cytometric analysis were less than 10%.

### S100B measurement

A commercially available enzyme-linked immunosorbent assay (ELISA) was used to measure S100B (Abcam, Cambridge, United Kingdom) in plasma. The analyses were conducted in accordance with the manufacturer’s protocols. The results are presented as positive values minus the background.

### Statistical analyses

To summarize demographic data, we used descriptive statistics with frequencies and percentages for categorial variables, and median with interquartile range (IQR). Levels of astrocyte-derived EVs and S100B are described in mean and SD. To investigate group-differences we used Independent Samples Mann-Whitney U test. All tests were considered significant if *p* < 0.05. Analyses were performed using the Statistical Package for Social Sciences version 28 (SPSS Inc., Illinois, USA), JMP software (SAS Institute, v17.0, Cary, North Carolina, USA) and Graphpad Prism (10.0, GraphPad Software Inc, La Jolla, CA).

## Results

In total, 90 children with JIA and 10 healthy children participated in the study. The vast majority were classified as oligoarticular persistent according to the ILAR critera [18]. The gender and age distribution did not differ significantly between the two groups with *p* = 0.22 for age. The level of disease activity was significantly higher in the group of children without medical treatment (*n* = 42) compared to those on treatment (*n* = 48), *p* = 0.009. The duration of disease at the time of sampling showed a wide variation (Table 1).

**Table 1:** Demographic data of the 90 children with JIA and 10 healthy children in the study

	Children with JIA *N* = 90			Healthy children *N* = 10
	Total group	No medical treatment *N* = 42 (46,7)	Medical treatment *N* = 48 (53.3)	
Gender, number of girls (%)	68 (69)			6 (60)
Age at onset, years, Md (IQR)	5.7 (2.5–11.8)			
Age at sampling, years, Md (IQR)	11.6 (6.7–15.1)*			8.4 (6.3–10.9)*
Disease duration at sampling	2.5 (0.9–5.8)	1.8 (0.2–5.1)	3.0 (1.6–6.7)	
JADAS27, Md (IQR)	4.2 (1.8–8.8)	6.3 (2.7–11.9)**	3.6 (0.7–5.7)**	
**ILAR category, n (%)**				
Oligoarticular persistent	60 (66.7)	31	29	
Polyarticular RF ^−^	15 (16.7)	5	10	
Juvenile psoriatic arthritis	8 (8.9)	4	4	
Oligoarticular extended	4 (4.4)	1	3	
Enthesitis-related arthritis	2 (2.2)	1	1	
Polyarticular RF^+^	1 (1.1)	0	1	
**Medical treatment, n (%)**				
MTX			27 (30)	
Biological agent + MTX			11 (12.2)	
Biological agent			10 (11.1)	

ILAR = International League of Associations for Rheumatology; RF = rheumatoid factor; MTX = metotrexat

*Independent-Samples Mann-Whitney U test, *p* = 0.22; ** Independent-Samples Mann-Whitney U test, *p* = 0.009

### Astrocyte-derived EVs

**Fig. 2:**
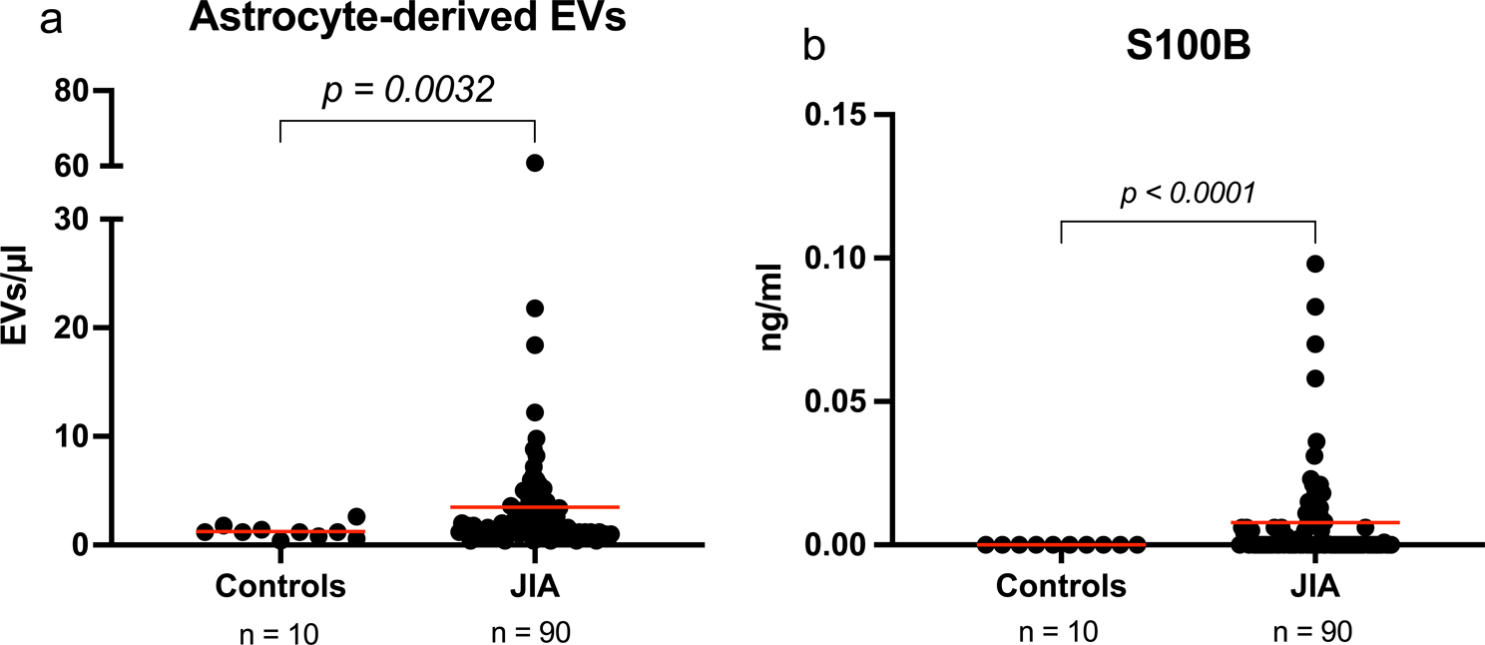
Analysis of astrocyte-derived extracellular vesicles and S100B levels in children with juvenile idiopathic arthritis (JIA) versus healthy controls. (**a**) Flow cytometric quantification of astrocyte-derived EVs expressing aquaporin-4 and glial fibrillary acidic protein. (**b**) S100B protein concentrations, determined by commercial ELISA. Red line indicates mean

Our flow cytometric analysis revealed a notable difference in the levels of astrocyte-derived EVs between children with JIA and healthy controls (Fig. 2a). The patient group had approximately 2.8 times higher values of astrocyte-derived EVs compared with controls (*p* = 0.0032, 1.2. ± 0.6 vs. 3.5 ± 6.9 EVs/µl respectively).

### S100B

Similar to astrocyte-derived EV results, the S100B concentrations measured by commercial ELISA, were significantly higher in JIA patients compared to controls (*p* < 0.0001, Fig. 2b). Notably, the control group did not have measurable values of S100B as compared with the patient group which demonstrated a mean value of 0.008 ± 0.02 ng/ml.

### Astrocyte-derived EVs and S100B & JADAS27

**Fig. 3:**
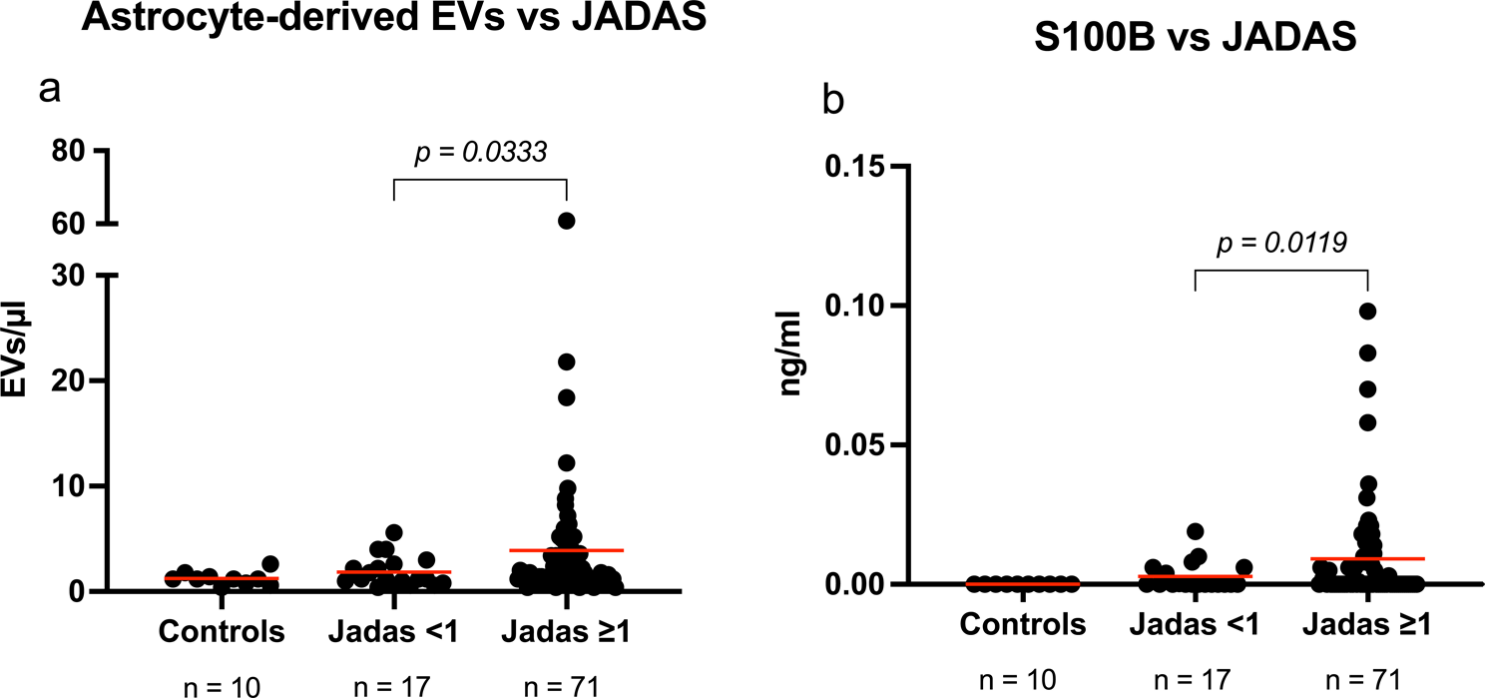
Relationship between CNS biomarkers and disease activity in juvenile idiopathic arthritis (JIA) patients, categorized by JADAS27 scores. (**a**) Analysis shows a significant increase in astrocyte-derived EV levels in patients with higher disease activity (JADAS27 scores above 1). (**b**) There is a comparable significant relationship for S100B protein concentrations, with higher disease activity (scores above 1) correlating with elevated levels. Red line indicates mean

In investigating the relationship between disease activity and neuroinflammatory markers in JIA, we analyzed the levels of astrocyte-derived EVs and S100B in patients categorized by their JADAS27 score (Fig. 3a and b). The results demonstrate a significant association between higher disease activity (as indicated by JADAS27 scores of 1 or higher) and increased levels of both astrocyte-derived EVs (Fig. 3a) and S100B (Fig. 3b).

For astrocyte-derived EVs, there was a notable increase in patients with higher JADAS27 scores (3.9 ± 7.6 vs. 1.8 ± 1.4 EVs/µl, *p* = 0.0333). Similarly, the concentration of S100B, was also significantly higher in the group with JADAS27 scores of 1 or higher (0.009 ± 0.02 vs. 0.002 ± 0.0005 ng/ml, *p* = 0.0119).

## Discussion

Our study presents important findings in the realm of juvenile idiopathic arthritis, expanding the understanding of its impact beyond the joints to the central nervous system. The elevated levels of astrocyte-derived EVs and S100B in JIA patients, as compared with healthy controls, underscore the potential involvement of the CNS in JIA pathology. This is especially notable given the limited prior research in this domain.

The significant increase in astrocyte-derived EVs measured in the blood of JIA patients indicates not only a profound change in astrocyte activity and potentially in intercellular communication within the CNS but also an impairment in BBB permeability. Astrocyte-derived EVs have been demonstrated to be elevated in several diseases linked to CNS functionality such as traumatic brain injury as well as stress-induced exhaustion disorder [16, 20]. The elevated levels in JIA patients could be indicative of a reactive or compensatory mechanism in response to systemic inflammation. This hypothesis is further supported by the significant association found between higher JADAS27 scores and increased levels of astrocyte-derived EVs and S100B, echoing similar findings in other inflammatory diseases. Moreover, studies have shown that systemic inflammation can lead to the formation of distinct populations of extracellular vesicles from microglia, which may play a role in cell-to-cell communication and the propagation of inflammatory signals in the CNS [15].

Interestingly, a recent study by Arnstad et al. found that fatigue is a prominent symptom in young adults with JIA, with higher fatigue burden after 18 years of disease among participants with a diagnostic delay ≥ 6 months. A disease course with a high burden of medication also predicted severe fatigue 18 years after onset [7]. The link between systemic inflammation, increased astrocyte-derived EVs, and BBB integrity suggests a possible connection between CNS involvement and the manifestation of fatigue in JIA patients. However, more studies are needed to elucidate this link and to understand the underlying mechanisms.

Another complication of JIA is the risk of persistent pain despite treatment, along with a lower pain threshold compared to healthy children. The alteration of peripheral pain processing pathways has been proposed as an explanation for this phenomenon [21]. A possible concurrent process could be the impact on the hippocampus, an area of the CNS sensitive to inflammation and involved in both pain regulation and mood regulation. In one study the structure of hippocampus area in children with JIA was found to be altered compared to controls [10]. However, in our study, we found no association between the degree of pain (VAS) and levels of astrocyte-derived EVs (data not shown).

It could also be speculated that the increased risk of mental comorbidity in JIA depends not only on the burden of a chronic disease with pain as a primary manifestation, but also on the influence of general inflammation on the CNS. In one study on adults with RA, peripheral inflammatory signals were transduced to central changes, patterns that predicted fatigue, pain and cognitive dysfunction. This support the notion that chronic inflammation affects the brain’s structure and function [22]. Cognitive dysfunction associated with the degree of inflammation has been demonstrated in adults with rheumatoid arthritis [23], but this has not been proven in children with JIA [24]. Han et al. studied brain metabolite levels in children with JIA and found evidence of persistent glial cell dysfunction, which supports our finding of astrocyte-derived EVs and S100B in peripheral blood [25]. Interestingly, the impaired astrocyte function persisted even in the inactive phase of JIA.

The role of S100B, a Ca2 + binding protein associated with astrocyte activity and BBB integrity, is particularly intriguing in the context of JIA. Our findings of significantly higher concentrations of S100B in JIA patients suggest a potential disruption or excess leakage of the BBB. S100B is abundantly expressed in the brain by astrocytes and has both autocrine and paracrine effects on neurons and glia [17].

Elevated levels of S100B have been observed in neurodegenerative diseases and brain injuries, such as Alzheimer’s Disease, Parkinson’s Disease, Down syndrome, and stroke, where it may act as a damage-associated molecular pattern (DAMP) protein [26, 27]. It has also been associated with chronic inflammation in conditions like rheumatoid arthritis, diabetes, and cystic fibrosis [28–30]. The role of S100B in these conditions is complex and may involve both pro-inflammatory and anti-inflammatory actions. In vitro studies have suggested that excessive production of S100B by astrocytes might lead to the production of pro-inflammatory mediators such as TNF-α and IL-1β by microglia, thus enhancing inflammation with mediators well known in JIA [31–33]. In the context of JIA, the elevated S100B levels could reflect a similar inflammatory process within the CNS. Given the role of S100B in enhancing inflammation via its action on macrophages and microglia, it is conceivable that S100B contributes to the pathophysiology of JIA by exacerbating CNS inflammation. This hypothesis is supported by our findings, which indicate a disruption of the BBB in JIA patients by two independent biomarkers of astrocytes (astrocyte-derived EVs and S100B).

The study’s limitations include its cross-sectional design, which precludes drawing causal inferences. Longitudinal studies are needed to elucidate the temporal dynamics between JIA activity, astrocyte-derived EVs, and S100B levels. Furthermore, the mechanisms underlying the elevated levels of these biomarkers in JIA patients remain to be fully understood. A limitation of our study was the relatively small size of our control group compared to the JIA samples; a challenge compounded by the substantial difficulties in recruiting healthy children for research that involves blood sampling. Despite this constraint, we ensured that all samples, both from controls and JIA patients, were handled and analyzed uniformly, maintaining consistency across our dataset. Additionally, our use of two independent methods to assess astrocyte biomarkers; EVs and S100B levels, allowed us to identify consistent differences between the patient group and controls. The time from sampling to centrifugation is also a limitation since the bioactivity in the tubes most likely have influenced the results. However, control samples were handled similarly to mitigate this effect. Additionally, the use of EDTA plasma instead of citrated plasma for EV measurements poses a limitation due to the potential for platelet activation and the subsequent formation of artefactual EVs [34]. However, since we didn’t specifically look for platelet markers, the issue of these artificial EVs should be minimal in our findings. Furthermore, both control and patient samples were collected and processed according to a standardized laboratory protocol, ensuring consistency in sample handling. Future research should focus on unraveling these mechanisms to develop targeted therapeutic strategies that address both joint and CNS manifestations of JIA.

In conclusion, our study sheds light on the previously underexplored area of CNS involvement in JIA. The findings highlight the importance of considering neurological health in JIA patients and open avenues for further research into the mechanisms and implications of CNS engagement in this disease.

### Electronic supplementary material

Below is the link to the electronic supplementary material.

Supplementary Material 1

## Data Availability

Datasets used and/or analysed during the current study are available from corresponding author on reasonable request.

## Abbreviations

EVs: extracellular vesicles
DAMP: damage-associated molecular pattern protein
ILAR: International League of Associations for Rheumatology
JADAS27: juvenile arthritis disease activity score
MTX: metotrexat
RF: rheumatoid factor
VAS: visual analogue scale
