# Supplementary material for: Blood brain barrier permeability and astrocyte-derived extracellular vesicles in children with juvenile idiopathic arthritis: a cross-sectional study
Source: Pediatr Rheumatol Online J. 2024 Apr 26;22:47. doi: 10.1186/s12969-024-00984-2 (PMC11046815; doi:10.1186/s12969-024-00984-2)
Supplement: Supplementary file 1 — Supplementary Material 1 [file 12969_2024_984_MOESM1_ESM.pdf]

Response to Reviewers

PROJ-D-24-00065

Thank you for the constructive feedback and helpful comments to further improve the manuscript.

Reviewer reports:

Reviewer #1:

The area of study covered in the paper by Berntson et al is certainly new and exciting. However, I have some concerns about the data presented. Although the level of astrocytes-derived EVs can reasonably be expected to be low in JIA, the sole representative Figure 1D barely shows stain for double positive AQP4/GFAP EVs. No other EV staining was presented, so it is not possible to evaluate if these low EV numbers are unique to the astrocytes-derived EVs or there are any other issues with the samples. The authors could at least show the levels of single AQP4+ EVs and single GFAP+ EVs in their samples, as done in Wallensten et al.

Authors: We appreciate your observation regarding the visual representation of double positive AQP4/GFAP EVs in Figure 1D. This figure indeed depicts raw data without adjustments for EVs/µl or consideration of the dilution factor, which might contribute to the perception of low event numbers.

It's important to emphasize that the detection of high levels of double positive EVs would be unusual and potentially alarming, suggesting significant BBB disruption. Our decision not to display single positive EVs was driven by our focus on astrocyte-specific markers, aiming to avoid potential misinterpretation from non-specific EV capture of molecules. We've implemented several controls, including the use of EV-free plasma, to ensure the accuracy of our measurements and minimize background noise. Moreover, all controls and JIA samples were analyzed concurrently, which minimizes variability and strengthens the significance of the differences observed between the control and JIA groups. We have added the clarification in the revised manuscript, please see page 7, line 158.

With such low EVs levels, it would have been useful to present further evidence about the astrocytes-derived EVs, perhaps using other astrocyte-related markers if available.

Authors: Recognizing the need for more evidence, we complemented our EV analysis with S100B measurements using a commercial ELISA, which is routinely employed in our hospital for assessing BBB integrity. This dual approach allowed us to explore different aspects of astrocyte activity and BBB condition, as S100B levels and EV production reflect different astrocyte. Importantly, when comparing S100B levels to NeuroEVs, we observed that these markers do not present a uniform picture, suggesting that they may represent mechanistically distinct aspects of astrocyte changes. Please see below figure demonstrating comparing NeuroEV levels to S100B concentration in JIA patients.

Another issue is the comparison between 90 JIA samples to only 10 control samples. With such a small control group, is the range of EVS in the control group adequately represented? While I am very aware of how difficult it is to collect samples from healthy children, it is still a concern to have a control group that is around 10% of the test group, especially without any other EV data.

Authors: We acknowledge the concern regarding the size of our control group in comparison to the JIA samples. While the recruitment of healthy children poses significant challenges, we understand the importance of a well-balanced control group for robust statistical analysis. This limitation has been carefully considered in the interpretation of our results and has now been addressed in the limitations section of our discussion. Please see page 13, line 288.

Considering the heterogeneity of their JIA sample, is there anything unique to the samples with the high levels of astrocyte-derived EVs? Are they the same samples as the ones with high levels of S100B?

Authors: Thank you for this comment. Our analysis did not reveal distinct clinical characteristics correlating with elevated levels of NeuroEVs or S100B among the patients. Interestingly, the presence of high levels of these markers did not coincide in the same patients (see below figure), suggesting that their release might be stimulated by different mechanisms – related to your previous comment. This divergence aligns with observations from other studies (Wallensten et al), indicating a complex interplay of factors influencing astrocyte activity and BBB integrity.

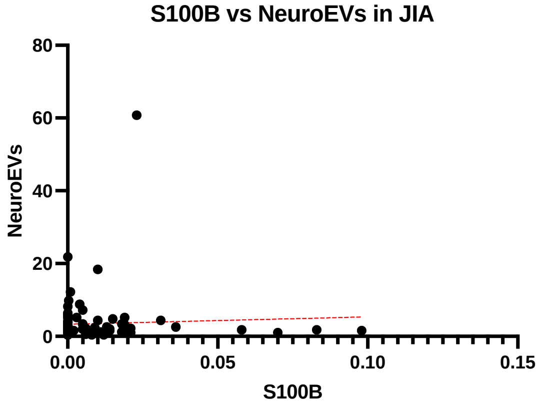

The authors show that the group with Jadas >1 has higher level of astrocyte-derived EVs compared to the Jadas <1 group, what is reassuring. How about treatment, does it affects the levels of astrocyte-derived EVs? The levels of S100B in the JIA group also support their hypothesis of BBB disturbance, but it does not really address the EV issues.

Authors: We investigated the impact of methotrexate treatment on NeuroEV levels and observed a trend towards higher levels in treated patients (methotrexate group), with a p-value of 0.041 (see below figure). No significant changes were noted in other groups or S100B levels. Given the complexity of interpreting these results and the potential for speculative discussion, we chose not to include these data in the manuscript. However, we find these initial observations compelling and believe they warrant further investigation.

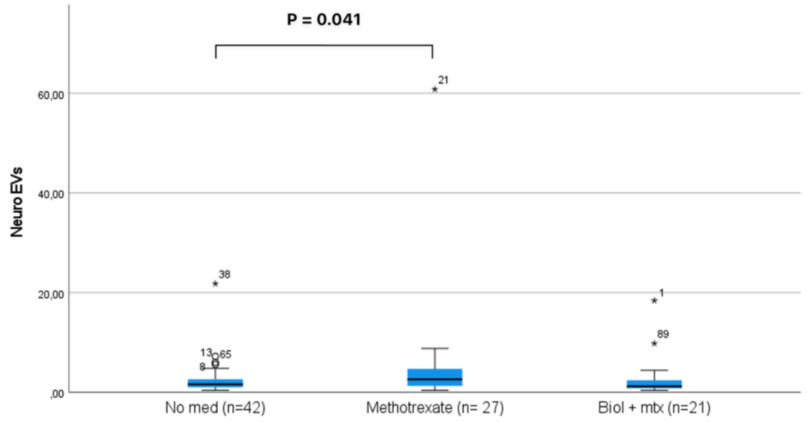

Samples: the plasma samples were frozen, but there is no description of sample thawing, please clarify.

Authors: All samples were thawed in water bath for approximately 5 min at 37°C prior to analysis. We have added this information in the Method section – please see page 7, line 139.

Reviewer #2:

Dear Authors,

I would like to congratulate the authors on their manuscript " Blood brain barrier permeability and astrocyte-derived extracellular vesicles in children with Juvenile Idiopathic arthritis: a cross-sectional study."

I have read your manuscript with interest and have some comments and suggestions.

Abstract:

No comments

Introduction:

No comments

Methods:

1. In the introduction it is mentioned development of pain amplification and fatigue can be one of the implications of the systemic inflammation on the brain. Could you explain why a fatigue measurement was not also performed?

Authors: At the time of designing our study and enrolling participants, the potential relationship between fatigue and its neurological implications in JIA was not widely recognized. Therefore, the omission of fatigue measurement was not a deliberate decision but rather a reflection of the state of knowledge and focus, at that time. Based on our results and the growing interest in the neurological aspects of JIA, we see the value of adding fatigue measurement in future studies.

2. I miss a power calculation for the 90 JIA patients and 10 controls. I assume these numbers are based on statistical power? if so it should be mentioned method section and if not based on a power calculations it should also be mentioned why these numbers where chosen (convenience sample etc) and should than be addressed in the discussion if this could have lead to confounding, bias etc.

Authors: Our study did not include a prior power calculation, primarily due to the logistical challenges in collecting samples from healthy pediatric subjects. The sample sizes were determined by the availability of participants willing to contribute to the present study. We acknowledge this as a limitation in our study design in our manuscript. Please see page 13, line 288.

Results:

1. It is mentioned disease activity is higher in non-treated patients compared to treated patients. And separately it is mentioned that higher disease activity leads to more neuoinflammatory markers. Did you also compared treatment or see if there was a potential treatment effect on neuroinflammatory markers?

Authors: Since reviewer 1 raised similar comment, we have included the same response and subsequent figure here for clarity:
We investigated the impact of methotrexate treatment on NeuroEV levels and observed a trend towards higher levels in treated patients (methotrexate group), with a p-value of 0.041 (see below figure). No significant changes were noted in other groups or S100B levels. Given the complexity of interpreting these results and the potential for speculative discussion, we chose not to include these data in the manuscript. However, we find these initial observations compelling and believe they warrant further investigation.

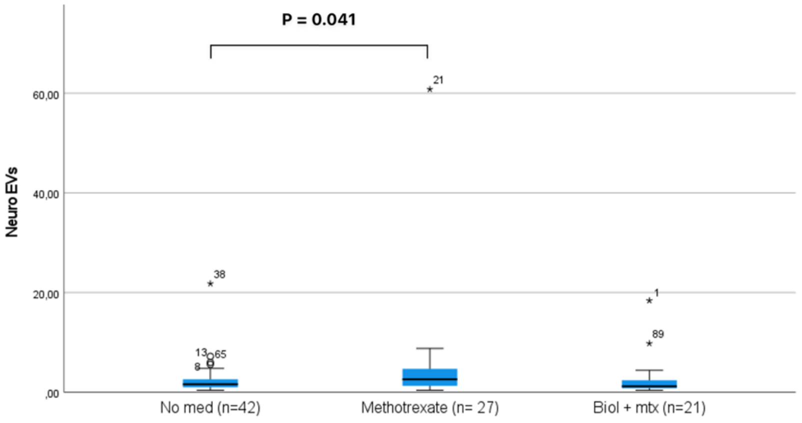

Discussion:

1. Please indicate in the discussion if the limited number of healthy controls could have lead to bias?

Authors: Yes, Please see page 13, line 288.

****
